# Tunable Fano Resonance in Asymmetric MIM Waveguide Structure

**DOI:** 10.3390/s17071494

**Published:** 2017-06-25

**Authors:** Xuefeng Zhao, Zhidong Zhang, Shubin Yan

**Affiliations:** Science and Technology on Electronic Test and Measurement Laboratory, North University of China, No. 3 Xueyuan Road, Taiyuan 030051, China; xf_zhao@st.nuc.edu.cn

**Keywords:** surface plasmon polaritons, refractive index, Fano resonance, finite element method

## Abstract

A plasmonic waveguide coupled system that uses a metal-insulator-metal (MIM) waveguide with two silver baffles and a coupled ring cavity is proposed in this study. The transmission properties of the plasmonic system were investigated using the finite element method. The simulation results show a Fano profile in the transmission spectrum, which was caused by the interaction of the broadband resonance of the Fabry-Perot (F-P) cavity and the narrow band resonance of the ring cavity. The Fabry-Perot (F-P) cavity in this case was formed by two silver baffles dividing the MIM waveguide. The maximum sensitivity of 718 nm/RIU and the maximum figure of merit of 4354 were achieved. Furthermore, the effects of the structural parameters of the F-P cavity and the ring cavity on the transmission properties of the plasmonic system were analyzed. The results can provide a guide for designing highly sensitive on-chip sensors based on surface plasmon polaritons.

## 1. Introduction

Surface plasmon polaritons (SPPs) are the charge-density waves caused by the coupling between electrons on the metal surface and photons [1,2,3]. Their electric fields decay exponentially in the direction perpendicular from the metal-dielectric interface [4,5,6]. As a result, SPPs can overcome the conventional optical diffraction limit and, therefore, are a promising technology in the realization of nanoscale optical manipulation, transmission, processing, and control [7,8,9]. In recent years, various photonic devices based on the SPP waveguide structures have been investigated and realized [10,11,12] such as biological and chemical sensors [13,14,15,16], filters [17,18], and all-optical switches [19].

Among the SPP waveguides, metal-insulator-metal (MIM) waveguides coupled with resonators are very popular and have captured the interest of researchers owing to the fact that they can confine light in deep-subwavelengths [20,21]. Therefore, many sensors using a MIM waveguide have been proposed and investigated. Zhang et al. [22] showed a symmetric plasmonic waveguide with a shoulder-coupled rectangle cavity that has a figure of merit of 57. Zhang et al. [23] also achieved a plasmonic refractive index nanosensor based on MIM waveguide-coupled double rectangular cavities, which have a refractive index sensitivity of 596 nm/RIU and a figure of merit of 7.5. Yun et al. [24] reported a MIM stub resonator coupled with a plasmonic square cavity resonator that has a refractive index sensitivity of 938 nm/RIU. Tang et al. [25] achieved a refractive index sensor based on a MIM waveguide coupled with a rectangular resonator and ring resonator, for which the figure of merit is 75. Among these, the resonator cavity has an important influence on the properties of the device. Therefore, the method of optimization of the plasmonic resonator is critical to the improvement of the sensitivity of the sensor. Recently, some novel plasmonic phenomena have been found in SPP waveguide systems, for instance, electromagnetically induced transparency [26], coupled-resonator-induced transparency [27], and Fano resonances [28]. Fano resonance is generated by the coherent coupling and interference between the discrete state and the continuous state [29,30]. Fano resonance exhibits a typically asymmetric and sharp line profile, which differs from the profile of the Lorentzian resonance [31]. At present, the use of plasmonic structures in Fano resonance-based sensors has become increasingly important in many fields. These fields include chemistry [32], physics [33], biology [34], energy, and information technology [35].

In this paper, a plasmonic waveguide system consisting of a MIM waveguide with two silver baffles and a ring resonator was studied numerically. Two silver baffles were used to obtain an F-P cavity in the MIM waveguide. The transmission spectra and magnetic Hz field distributions were calculated using the finite element method (FEM) with a perfectly matched layer absorbing boundary condition. The influence of the structural parameters of the plasmonic waveguide system on the Fano resonance was investigated. Furthermore, the shifts in the Fano resonance dip with the different refractive indexes of the filled dielectrics were studied.

## 2. Structural Model and Analytical Method

**Figure 1:**
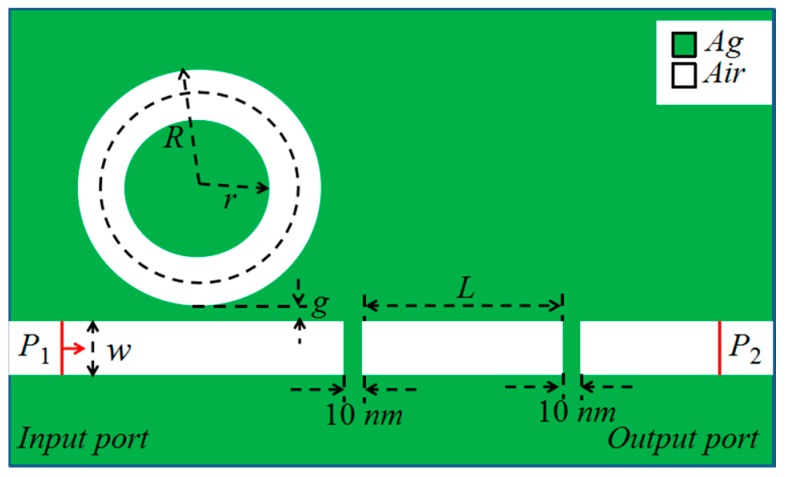
Schematic diagram of the plasmonic waveguide coupling structure.

A basic schematic of the proposed plasmonic waveguide coupling system is illustrated in Figure 1. The proposed system is composed of a MIM waveguide with two silver baffles and a ring cavity. The white areas and green areas represent air and silver, respectively. In all simulations performed in this study using FEM, the relative permittivity of silver is described by the Debye-Drude dispersion model [36]:

(1) $$\varepsilon \left(\omega \right)=\varepsilon_{\infty }+(\varepsilon_{s}-\varepsilon_{\infty })/(1+i\omega \tau )+\sigma /i\omega \varepsilon_{0}$$

where *ε*_∞_ = 3.8344 is the permittivity of infinite frequency, *ε_s_* = −9530.5 is the plasma frequency corresponding to the frequency of oscillations of the free electrons, and *τ* = 7.35 × 10^−15^ is the relaxation time.

The outer and inner radii of the ring cavity are expressed as *R* and *r*, respectively. *L* is the length of the F-P cavity. The MIM waveguide is separated by two silver baffles with their widths fixed at 10 nm. The ring cavity is side-coupled to the F-P cavity. The coupling distance is *g*. The widths of the MIM waveguide and the ring cavity are fixed at *w* = 50 nm. Therefore, the MIM waveguide only supports the fundamental transverse magnetic (TM_0_) modes [37]. The transmission direction is from the input port (*P*_1_) to the output port (*P*_2_). The transmittance can be expressed as *T =* (S_21_)^2^, where S_21_ is the transmission coefficient from *P*_1_ to *P*_2_ [23].

## 3. Results and Discussion

**Figure 2:**
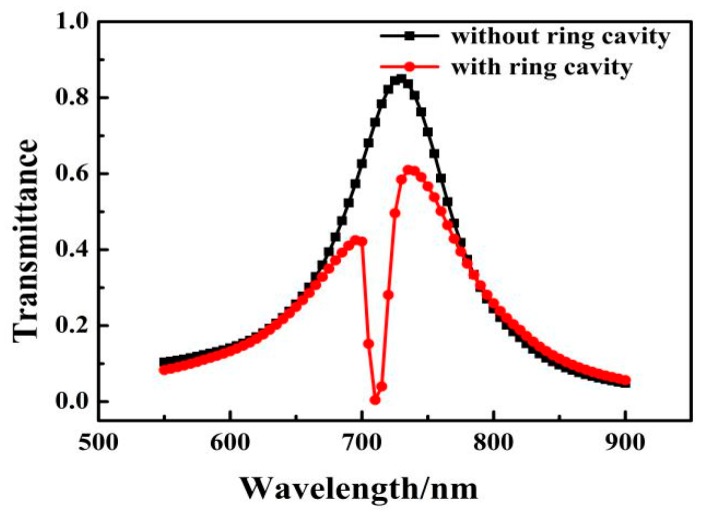
Transmission spectrum of the MIM waveguide without the ring cavity and with a side-coupled ring cavity.

To better understand the transmission properties of the proposed structure, we constructed MIM structures without the ring cavity and with the ring cavity. The transmission spectra of the MIM waveguide without the ring cavity and the MIM waveguide with a side-coupled ring cavity are shown in Figure 2 as a black curve and a red curve, respectively. The initial values of the parameters are *L* = 200 nm, *n* = 1 RIU, *r* = 134 nm, and *g* = 10 nm. The spectral line in the MIM waveguide without the ring cavity is similar to a Lorentz line. However, in the MIM waveguide with a side-coupled ring cavity, the transmission spectrum displays an obvious split at *λ* = 710 nm, which is known as the Fano resonance.

**Figure 3:**
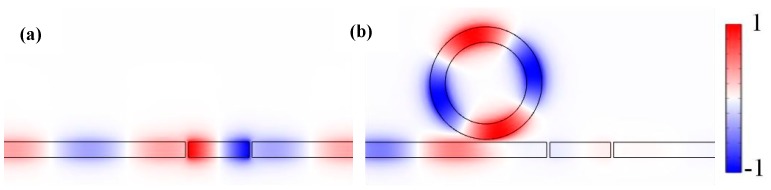
Contrast diagram of the normalized magnetic field distribution of the MIM waveguide at *λ* = 710 nm (**a**) with the ring cavity and; (**b**) without the ring cavity.

The *H_z_* field distributions with the incident wavelength of 710 nm were calculated to analyze the internal mechanism of the spectrum split. As shown in Figure 3a, an obvious resonance was created in the F-P cavity. However, Figure 3b shows that a weak resonance was formed in the F-P cavity when a ring cavity was added to the structure. The analysis shows that the Fano resonance is caused by the destructive interference between the broadband resonance of the F-P cavity and the narrow band resonance of the ring cavity [38].

**Figure 4:**
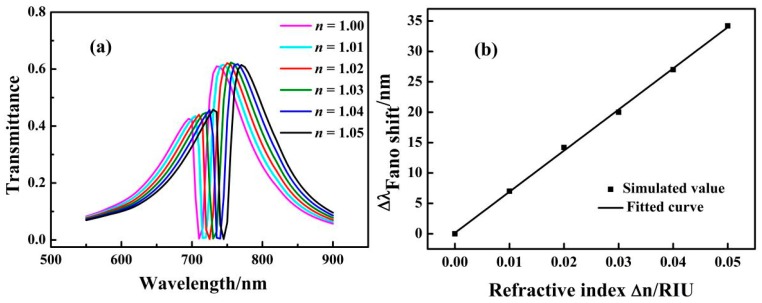
(**a**) Transmission spectra for different refractive indexes *n*; (**b**) Fitting curve of Fano resonance peak shift caused by the change in the refractive index of *Δn*.

The transmission spectra were simulated using different filling media to investigate the effect of the refractive index (*n*) on the structure. Figure 4a shows that the transmission spectra of the refractive index increases from 1 to 1.05 RIU at intervals of 0.01 RIU. The simulation results showed that the transmission spectrum exhibited a red shift with an increase in *n*. In this study, we employ the figure of merit (FOM) and sensitivity to calculate the sensitivity properties. FOM is a key parameter for the nanosensor. It can be defined as [39]:

(2) $$FOM=\frac{\Delta T}{T\Delta n}$$

where *T* is the ultra-low transmittance and *ΔT/Δn* is the change rate of the transmittance caused by the change in the refractive index. As shown in Figure 4b, the solid line is obtained by linear fitting. The sensitivity (*Δλ/Δn*) is 677 nm/RIU, and its FOM is 1795.

**Figure 5:**
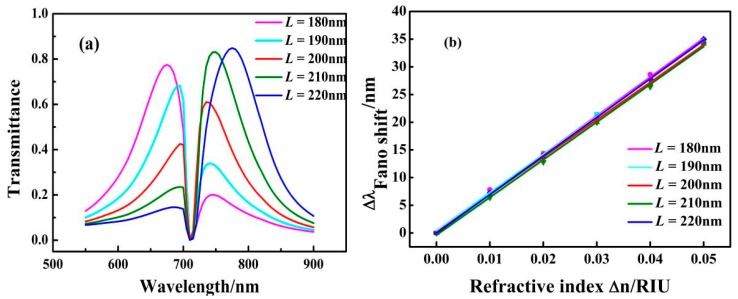
(**a**) Transmission spectra for different lengths *L* of the coupling cavity; (**b**) Fitting curves of Fano resonance peak shift caused by a change in the refractive index of *Δn* with different values of *L*.

For the investigation of the effects of different lengths of the F-P cavity on the Fano resonance of the MIM waveguide, *L* was increased from 180 nm to 220 nm in steps of 10 nm, keeping other parameters fixed at *n* = 1 RIU, *r* = 134 nm, and *g* = 10 nm. The transmission spectra are shown in Figure 5a. With increasing *L*, the resonance peak transmittance on the left side of the dip shows an obvious decrease and the resonance peak transmittance on the right side of the dip shows an obvious increase. Moreover, the position of the dip did not shift. This phenomenon can be explained by the fact that an increase in *L* caused a red shift in the broad spectrum of the F-P cavity and the position of dip is determined by the resonance of the ring cavity. As shown in Figure 5b, the solid lines are obtained by linear fitting. The sensitivity changes slightly with the increase in length *L* of the F-P cavity. This illustrates that the sensitivity can maintain its high value when the structure is adjusted. From calculations, the maximum sensitivity of the structure with *L* = 180 nm is 698 nm/RIU, and the maximum FOM is 4354.

**Figure 6:**
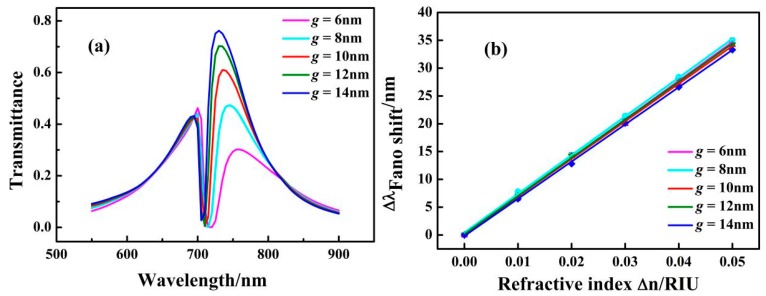
(**a**) Transmission spectrum for different versus of the coupling distance *g*; (**b**) Fitting curve of Fano resonance peak shift caused by the refractive index change of *Δn* with different values of *g*.

The coupling distance between the ring cavity and the F-P cavity was changed to study its effect on the transmission properties. The coupling distance g was increased from 6 nm to 14 nm while other parameters were fixed at *n* = 1 RIU, *r* = 134 nm, and *L* = 200 nm. The transmission spectra of the structure with different distances of the ring cavity and the F-P cavity for *g* = 6, 8, 10, 12, and 14 nm are shown in Figure 6a. With increasing *g*, the resonance peak transmittance on the left side of the dip decreases slightly and the resonance peak transmittance of the right side shows an obvious increase. Simultaneously, the position of the dip has a tiny blue shift. The phenomenon can be explained by the fact that the coupling distance changes the width of the narrow spectrum to some extent. When the coupling distance was expanded, the narrow spectrum narrowed further, resulting in a blue shift of the center. The solid lines shown in Figure 6b are obtained by linear fitting. The maximum sensitivity of the structure with *g* = 8 nm is 697 nm/RIU, and the value of the maximum FOM is 2693.

**Figure 7:**
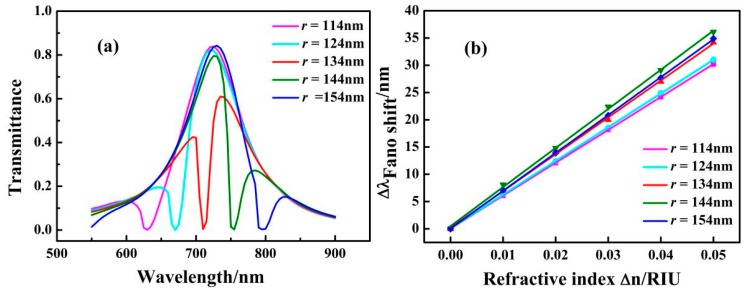
(**a**) Transmission spectra for different values of the inner radius of the ring cavity *r*; (**b**) Fitting curves of Fano resonance peak shift caused by the change in the refractive index of *Δn* with different values of *r*.

The influences of the different ring cavities on the transmission spectra of the MIM structure were investigated. The inner radius of the ring cavity *r* was increased from 114 to 154 nm in steps of 10 nm with the other parameters fixed at *n* = 1 RIU, *g* = 10 nm, and *L* = 200 nm. The transmission spectra of the structure with the different ring cavities of *r* = 114, 124, 134, 144, and 154 nm are shown in Figure 7a. With increasing *r*, the Fano resonant dip shows a redshift at equal intervals, and the line shape of the transmittance spectrum shows a symmetric distribution for *r* = 114 nm to *r* = 154 nm. This phenomenon can be explained by the fact that the position of the dip is determined by the narrow spectrum, and the narrow spectrum broadens with increasing *r*. The increase of *r* leads to the increase in the narrow band resonant wavelength, which causes the red shift of the Fano resonance. Figure 7b shows the solid lines obtained by linear fitting. The maximum sensitivity of the structure with *r* = 144 nm is 718 nm/RIU, and the value of the maximum FOM is 2025.

## 4. Conclusions

The transmission properties of the MIM waveguide with two silver baffles and a coupled ring cavity were studied using FEM. An asymmetric Fano resonance profile was observed in the transmission spectrum, and it was caused by the destructive interaction between the broadband resonance of the F-P cavity and the narrow band resonance of the ring cavity. The position of the Fano resonance dip and the sensitivity remained almost unchanged with increasing length of the F-P cavity and the coupling distance between the ring cavity and the F-P cavity. With increasing inner radius of the ring cavity, the Fano resonance dip showed a red shift and the sensitivity increased initially and then decreased. The simulation results show that the maximum sensitivity of 718 nm/RIU and FOM of 4354 were achieved. The proposed structure has potential applications in optical communication devices. Moreover, it can be conveniently integrated with other chip-scale photonic devices.
